# Female Reproductive Aging and Oxidative Stress: Mesenchymal Stem Cell Conditioned Medium as a Promising Antioxidant

**DOI:** 10.3390/ijms24055053

**Published:** 2023-03-06

**Authors:** Kihae Ra, Se Chang Park, Byeong Chun Lee

**Affiliations:** 1 Department of Theriogenology and Biotechnology, College of Veterinary Medicine, Seoul National University, Seoul 08826, Republic of Korea; 2 Laboratory of Aquatic Biomedicine, College of Veterinary Medicine and Research Institute for Veterinary Science, Seoul National University, Seoul 08826, Republic of Korea

**Keywords:** antioxidant, assisted reproductive technologies, female reproduction, infertility, mesenchymal stem cell, oxidative stress, reproductive aging, stem cell conditioned medium

## Abstract

The recent tendency to delay pregnancy has increased the incidence of age-related infertility, as female reproductive competence decreases with aging. Along with aging, a lowered capacity of antioxidant defense causes a loss of normal function in the ovaries and uterus due to oxidative damage. Therefore, advancements have been made in assisted reproduction to resolve infertility caused by reproductive aging and oxidative stress, following an emphasis on their use. The application of mesenchymal stem cells (MSCs) with intensive antioxidative properties has been extensively validated as a regenerative therapy, and proceeding from original cell therapy, the therapeutic effects of stem cell conditioned medium (CM) containing paracrine factors secreted during cell culture have been reported to be as effective as that of direct treatment of source cells. In this review, we summarized the current understanding of female reproductive aging and oxidative stress and present MSC-CM, which could be developed as a promising antioxidant intervention for assisted reproductive technology.

## 1. Introduction

For the success of reproduction, normal functioning of the ovaries and uterus is essential in both natural fertilization and in vitro fertilization (IVF). First, from a healthy ovary, oocytes are matured and ovulated with the appropriate quantity and quality [1]. Then, the oocytes are inseminated with sperm, and the fertilized zygotes are developed in fallopian tubes or under in vitro culture conditions. Both in vitro and in vivo developed embryos should be implanted, which means attaching to uterine tissue, and, finally, the pregnancy should be maintained with a receptive uterus [2]. However, aging leads to various abnormal phenotypes and dysfunctions in the ovaries and uterus, which increases age-related reproductive disorders [3]. Age-related infertility decreases the success rate of assisted reproduction, especially IVF, resulting from reproductive disorders such as polycystic ovarian syndrome [4], endometriosis [5], and other uterine diseases [6].

**Figure 1:**
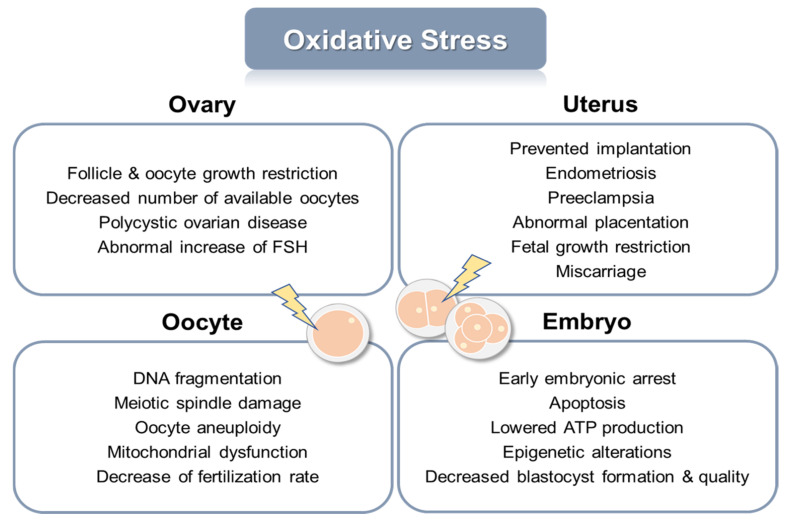
Detrimental effects of oxidative stress on female reproductive organs, oocytes, and embryos.

Reactive oxygen species (ROS) are essential regulators of various cellular processes, and, in a normal state, the number of free radicals and antioxidants are in balance [7]. However, when excessive free radicals are produced, the antioxidant defense fails, and healthy cells are damaged by oxidative stress [8]. Oxidative stress leads to mitochondrial damage [9], protein oxidation [10], lipid peroxidation [11], and DNA damage and mutation [12], all of which deteriorate biological functions and finally trigger the aging of cells [13]. Interactively, the incidence of oxidative damage also increases with aging [14]. It is widely acknowledged that oxidative stress brings detrimental consequences to the ovaries, uterus, oocytes, and embryos (Figure 1). In the ovaries, oxidative stress restricts follicle and oocyte maturation, which leads to a decreased number and competence of oocytes and an abnormal increase of the follicle-stimulating hormone [15,16,17]. In the uterus, implantation is prevented, and pregnancy complications arise, such as endometriosis [18], pre-eclampsia [19], and placentation failure [20], finally leading to fetal growth restriction and miscarriage [21]. Oxidative damage to the oocytes causes DNA fragmentation, mitochondrial dysfunction, aneuploidy, and, accordingly, a decrease in the fertilization rate [22,23,24]. Even after embryos are produced, oxidative stress can cause their apoptosis [25] and a decrease in the quantity and quality of blastocysts [26]. In general, oxidative stress on embryos developed in vivo is caused by aging and other environmental factors [27]. There are an additional number of stressors for embryos in the case of in vitro production, such as oxygen concentration, visible light, pH, temperature, and, especially, suboptimal culture medium, which significantly impairs the developmental competence of embryos [28,29,30]. Consequently, these challenging circumstances, associated with numerous factors for oxidative stress to which embryos are exposed, are driving the need to evolve the efficient application of antioxidants for the improvement of female reproductive competence and embryo development.

Mesenchymal stem cells (MSCs) can be ethically obtained from various sources, and they are safe, easy to harvest, and highly available [31,32]; therefore, they have been studied as regenerative medicine, and their therapeutic effects are widely known [33]. In particular, the potential of MSCs to alleviate oxidative damage caused by an array of diverse diseases has recently received significant attention. Stem cell conditioned medium (CM) was developed following the direct administration of stem cells. Comparing MSCs and MSC-CM, MSC-CM is considered more advantageous in terms of safety and convenience [34]. The therapeutic effect of CM is known to be comparable to stem cells themselves as paracrine factors, which are growth factors and cytokines [35], are secreted into the culture medium by stem cells, and these secretory factors are then present in the CM [36]. The purpose of this review is to summarize the impact of aging and oxidative stress on female reproduction and to discuss the recent findings of MSCs and MSC-CM, which are promising antioxidants.

## 2. Reproductive Aging 

Aging is defined as the age-related deterioration of the physiological functions required for the survival and fertility of all individuals of a species [37]. In particular, female reproductive competence decreases with aging, which has distinct implications in light of the contemporary trend to postpone childbearing following the development of contraceptive methods and the global flow of social and economic changes. Women of advanced maternal age (AMA) who get pregnant at the age of 35 or over have a risk of adverse obstetric outcomes such as miscarriage, low birth weight, pre- or post-term delivery, pre-eclampsia, gestational hypertension, neonates that are small or large for gestational age, and cesarean delivery [38,39,40]. Due to the general tendencies stated above, an increasing number of women attempting to conceive are experiencing female infertility and consequently depend on assisted reproductive technology (ART). Considering the importance of oocyte quality and embryo implantation in the process of unassisted reproduction, the condition of the ovaries and uterus in aged females should be considered in order to increase the success rate of ART [41].

### 2.1. Aging of the Ovaries

The ovaries are the most seriously affected by aging among the types of tissues in the female body, which leads to a negative correlation between age and reproductive capacity [42]. The general definition of physiologic ovarian aging is age-dependent declines of the functional ovarian reserve within expected ranges, which determines the loss of female fertility [43]. Primordial follicles are formed during fetal life and contain immature oocytes until puberty starts and then reach maturity in adult females [44]. Follicular atresia occurs until reproductive senescence, and this becomes progressively accelerated in the late reproductive period before menopause [45]. The decrease in follicles with aging gives rise to the elevation of follicular stimulation hormone (FSH), a stimulant currently used for ovarian stimulation and follicular development in assisted reproduction [46]. Increased levels of FSH results in the maturation of granulosa cells becoming unsynchronized with premature oocytes, which causes follicular atresia and apoptosis [42]. In addition to FSH, inhibin-B and anti-Mullerian hormone are recognized as sensitive markers of ovarian aging, with respect to the follicle population, which can be used to predict the results of ART [47]. 

As aging progresses, the quality of oocytes—the main determinant of embryo developmental competence—deteriorates, along with a gradual decrease in the ovarian follicular pool. Genetic defects in aged oocytes, which eventually affect female fertility, have been revealed as follows: Deletion of mitochondrial DNA [48], decrease in antioxidant enzymes [49], and facilitation of apoptosis in oocytes [50], observed in studies of granulosa cells from aged women. Compared to young oocytes, old oocytes exhibit mitochondrial dysfunction that results in abnormal calcium oscillation signals [51], spindle formation [52], and chromosomal alignment [53]. In particular, the high incidence of aneuploidy, which is the leading cause of spontaneous abortion and fecundity decline, has been found in oocytes and preimplantation embryos from aged women [54]. The activity of telomerase also decreases with aging, which could damage the intact chromosomes in oocytes [55]. Regarding the microenvironment, the production of ROS increases during aging and affects the outcomes of ovarian aging. The correlation between oxidative stress and ovarian aging is associated with abnormal mitochondrial functions and mutations in oocytes whose antioxidant defense weakens with aging [56,57,58]. In fact, mitochondrial antioxidant gene expression is diminished in aged oocytes in mice [59]. This is not only true of mitochondria; it is also known that oxidative stress in lipids, proteins, enzymes, and DNA in aged oocytes causes the deterioration of the quality of the oocytes [22]. In summary, ovarian aging can be characterized as a decrease in follicular recruitment, number of follicles, oocyte, and embryo quality, and available numbers of oocytes and embryos for ART and pregnancy rates with an increase in aneuploidy and poorly qualified oocytes [60].

### 2.2. Aging of the Uterus

The uterus is a reproductive organ that acts as an essential site for the process of pregnancy—from embryonic development, through implantation and placentation, to fetal development. Nevertheless, the effect of uterine factors on age-related infertility is uncertain in contrast to accumulating evidence showing the impact of ovarian aging on female fertility [61]. The irrelevance of uterine aging to reproductive competence has been assisted by a report that when embryos are transferred using oocytes from a young donor, the rate of implantation is not affected by the age of the recipients, and the uterus of aged recipients is capable of supporting an early pregnancy [62]. However, the results of studies on young oocyte and old uterine recipients are controversial in that the implantation rates are similar, regardless of the recipient’s age after in vitro fertilized embryos of equivalent quality are transferred; however, the rate of pregnancy loss increases in older recipients [63]. 

The uterus is an aging organ required to support a developing fetus during pregnancy. Aging of the uterus is associated with a decline in the sensitivity to hormones such as progesterone and endometrial receptivity with compromised uterine blood flow, with an increase in the occurrence of uterine fibroids and endometrial polyps [64]. In addition, aging of the uterus downregulates the genes involved in cell proliferation in mice, indicating impaired proliferation in aged uterine cells [65]. On the contrary, age-related upregulation of the immune and inflammatory response pathway has been observed in rat uterine horns [66], and inflammation and interferon-signaling pathways are activated in aged bovine endometrial cells [67]. Furthermore, inadequate placentation, which occurs in aged uteruses, is concerning, with complications in late pregnancy such as miscarriage and fetal growth restriction [68]. Increasing reports indicate that the competence of the uterus is influenced by aging, which could lead to infertility and reproductive complications, especially when considering pregnancy.

## 3. Oxidative Stress in Female Reproduction

Biological ROS, including superoxide anions, hydroxyl radicals, peroxyl, alkoxyl, and hydroperoxyl, are produced by incomplete reduction of oxygen in the process of mitochondrial oxidative metabolism and cellular responses to exogenous sources [69]. ROS are known as essential regulators that activate and modulate various signaling pathways, including those involved in cell growth, differentiation, metabolism, and apoptosis [70]. Even though ROS plays a crucial role in human physiological and pathophysiological processes as signaling molecules, cellular damage, and dysfunction can be induced by ROS at immoderate concentrations [71]. 

Any substance that substantially inhibits the oxidation of the oxidizable substrate can be defined as an antioxidant [72]. In general, endogenous antioxidants are classified into enzymatic and non-enzymatic antioxidants. Superoxide dismutase (SOD), glutathione peroxidase (GPx), and catalase (CAT), all of which are enzymatic antioxidants, collectively function in the primary defense mechanism to neutralize free radicals and prevent their additional formation [73]. In detail, SOD catalyzes superoxide anion dismutation to produce hydrogen peroxide and oxygen, and then CAT removes excessive hydrogen peroxide. Assisted by glutathione (GSH) as a co-substrate, GPx catalyzes the reduction of hydrogen peroxide to water and oxygen [74]. Non-enzymatic antioxidants are involved in the primary defense against ROS but also act as a secondary defense by scavenging free radicals from the antioxidants, imparting from their electrons, resulting in harmless radicals [73,75]. Glutathione is the most abundant non-enzymatic antioxidant synthesized in cells, which preserves cells from oxidative damage and maintains redox homeostasis [76]. It is also engaged in the repair processes of protein molecules, nucleic acids, and lipids in cells damaged in peroxidation processes [77].

Cells in a normal state have a defense system against oxidative stress, sustained by an appropriate balance between ROS and antioxidants; however, oxidative stress occurs when an excessive amount of ROS are produced or antioxidants’ defense capacity is weakened, finally resulting in their imbalance [78]. Oxidative stress impairs essential cell signaling cascades, and it accumulates with aging [79]. Reversely, the oxidative stress theory of aging has been proposed, stating that the age-associated loss of biological functions is due to the accumulation of oxidative damage to lipids, DNA, and proteins [80]. However, besides aging, a variety of endogenous and exogenous factors together contribute to oxidative stress. 

### 3.1. Oxidative Stress in the Ovaries and Uterus

Both positive and negative effects are brought about by ROS in mammalian ovaries. ROS affects various physiological and pathological functions of the ovaries, such as ovarian steroid genesis, follicular growth, oocyte maturation, ovulation, fertilization, implantation, and luteal maintenance in pregnancy [74]. In the ovaries, ROS generated during an inflammatory reaction from macrophages, leukocytes, and cytokines in the follicular fluid induce oocyte maturation and then follicle rupture, all required for ovulation [17,81]. Therefore, ROS produced at an appropriate level by preovulatory follicles are critical inducers of ovulation processes, which is supported by a report that the inhibition of ROS using enzymatic antioxidants suppresses ovulation [82]. However, excessive ROS leading to oxidative stress involves abnormalities in female reproduction, representing premature ovarian failure (POF) and polycystic ovary syndrome (PCOS). The early cessation of menstruation before the age of 40, known as POF, is associated with prematurely injured ovarian function due to impaired development or abnormal depletion of follicles as a result of accelerated apoptosis caused by high ROS levels [83]. One of the most prevalent reproductive disorders, PCOS, is characterized by hyperandrogenism, ovulatory dysfunction, and polycystic ovaries [84]. In contrast, a decline of total antioxidant levels and an elevation of oxidative stress in PCOS patients affect the abnormal formation of cysts and ovarian extracellular remodeling, which leads to anovulation and infertility [85]. 

After ovulation, ROS are generated in the corpus luteum, which plays a major role in progesterone synthesis [86]. In the endometrium, progesterone is essential for the development of the uterine environment in that it modulates growth factors, cytokines, proteins, and other hormones—the critical regulators of conceptus implantation, survival, growth, and, finally, the progression of pregnancy [87]. Nevertheless, when ROS are produced excessively, oxidative damage to the corpus luteum impairs the generation of progesterone, which can be detrimental to embryo development and pregnancy maintenance [88]. 

In the uterus, responsible for proper ROS function levels in angiogenesis, regeneration of the endometrium during reproductive cycles, and continuance of pregnancy unless imbalanced, oxidative stress influences the structure and functions of the uterus, including endometrial shedding and prevention of implantation [89]. During pregnancy, oxidative stress can lead to early pregnancy loss, as implantation of the embryo may be impeded, and normal immune functioning may be altered in the uterus [74]. Oxidative stress from damaged antioxidant systems interrupts the decidualization of the endometrium, which is critical for successful implantation [90], and pre-eclampsia and endometriosis caused by endothelial cell dysfunction can occur [91]. Pre-eclampsia, a hypertension disorder with maternal and neonatal mortality if seriously progressed, is also connected to abnormal placentation [92]. The environment of the pregnant uterus influences placental development, and oxidative stress induced in the placenta is associated with intrauterine fetal growth restriction and miscarriage [93].

### 3.2. Oxidative Stress in the In Vitro Production of Embryos

An embryo is a rapidly developing organism with an energy demand, which is supplied by ATP produced through mitochondrial oxidative phosphorylation and glycolysis, leading to the continuous modulation of a redox state. In particular, in the developmental processes of an embryo, the generation of ROS surges at points of embryonic genome activation, embryonic compaction, and hatching [28]. The balance of ROS and antioxidants is maintained at physiologically normal levels in in vivo female reproductive systems. On the contrary, protection is lost when fertilization is attempted outside the reproductive system in in vitro circumstances, resulting in an increase in exposure to oxidative risk [94]. During ART, numerous external factors generating oxidative stress are present from the technique of ART to environmental sources, such as oxygen concentration, temperature, visible light, pollutants, media, and supplements [28]. Excessive levels of ROS have a deleterious impact on embryo quality and subsequently block or delay early embryonic development [95]. In fact, an increase in ROS in embryos cultured under in vitro conditions can be connected to negative outcomes of ART, including embryo development and, lastly, pregnancies compared to in vivo [96]. 

To reduce oxidative stress during the in vitro production of embryos, the temperature of gametes and embryo incubation is recommended to be controlled as close to that of the in vivo state as possible, as an improper temperature could cause heat stress-induced oxidative damage [97]. The oxygen concentration provided for embryo incubation is more appropriate at a reduced level (5%) than at an atmospheric level (20%) to prevent the generation of ROS and consequent oxidative stress [98]. Among others, the application of antioxidants in procedures using ART can be an effective intervention to counteract oxidative damage to gametes and embryos, considering that the composition of culture media can directly affect the quality of in vitro-produced embryos and the success rate of ART [99]. For this reason, a number of publications have demonstrated their findings with respect to the beneficial effects of antioxidant supplementation on the embryo culture medium (Table 1). In addition to the antioxidant compounds above, cytokines and growth factors can also be treated in the embryo culture medium [100,101,102,103].

## 4. Mesenchymal Stem Cells

Stem cells are defined as cells with the ability to self-renew and can differentiate into specialized cells with multilineages in a controlled manner [111]. Stem cells are generally categorized into embryonic, adult, and induced pluripotent stem cells. Even though the biological and clinical importance of stem cells has been recognized for decades, limitations exist with respect to the ethical issues of embryonic stem cells and the risk of teratoma formation of induced pluripotent stem cells [112]. Accordingly, adult MSCs, first named by their ability to differentiate into lineages of cells developed from mesoderm, have received attention in that MSCs are multipotent, highly accessible, and available in in vitro cultures with genomic stability, and are safe from ethical issues, all of which emphasize the importance of MSCs as a regenerative medicine [113]. The International Society for Cellular Therapy clarified the standard for the characterization of MSCs as follows: (1) Plastic adherence and morphology resembling fibroblasts, (2) multipotential and multilineage differentiation ability, and (3) expression of specific phenotypic markers, including a cluster of differentiation (CD) 73, CD90, and CD 105 but not lineage-specific markers such as CD14, CD34, and CD45 [114]. The treatment of MSCs is known to be effective on autoimmune, inflammatory, and degenerative diseases, with its therapeutic mechanisms including homing efficiency to the damaged target site, differentiation ability, availability of tissue-engineered manipulation, immunomodulation, and abundant production of paracrine signaling factors [112]. Over the past few decades, research on MSCs has been actively developed, resulting in more than half a million publications, and their medical potential has been explored in approximately a thousand clinical trials registered with the United States Food and Drug Administration [115].

Present in most of the tissues in the body, MSCs have been isolated from multiple tissues such as bone marrow [116], skeletal muscle [117], umbilical blood [118], dental pulp [119], peripheral blood [120], synovium [121], adipose tissue [122], amniotic fluid [123], articular cartilage [124], placenta [125], lung [126], umbilical cord [127], and amniotic membrane [128]. Among these, MSCs derived from bone marrow (BMSCs), and adipose tissue (ASCs) are the most representative and verified with their regenerative ability [129]. Both BMSCs and ASCs possess the strong potential of in vitro differentiation into osteocytes, chondrocytes, adipocytes, hepatocytes, cardiomyocytes, pancreatic cells, and neuronal cells, but the differential differentiation capacities between them have only recently been found [130]. Compared to BMSCs isolated by an invasive surgical procedure with a certain level of pain and danger, ASCs are isolated from subcutaneous liposuction with greater ease and less pain, and 100–500-fold more stem cells can be harvested from adipose tissue than bone marrow [131]. It is widely considered that ASCs are effective regenerative medicine for the treatment of numerous symptoms, including pathological wound healing, refractory acute graft-versus-host disease, and hematologic and immunological disorders [132]. Most of all, the prominent property of ASCs is their antioxidative effect on various diseases and species [133,134,135,136]. Meanwhile, MSCs derived from the amniotic membrane (AMSCs) have emerged prospectively in the field of regenerative medicine due to their simple and abundant acquisition, reduced damage of donor, multipotency, low immune response, and the minimal ethical issue associated with their use [137]. The amniotic membrane is a component of the placenta that protects the fetus during pregnancy, with its structure of thick collagen layers and function of nutrient supplementation, but it is generally discarded post-partum and infrequently utilized compared to other types of MSCs [138]. To date, the amniotic membrane is a relatively new source of MSCs and has been identified as having anti-inflammatory, antiangiogenic, and anti-immunogenic properties [137,138]. In addition, AMSCs have the capability of differentiating all three germ layers, including ectodermal lineage cells, mesodermal lineage cells, and endodermal lineage cells [139], reiterating their value as a recommendable candidate for novel regenerative medicine. 

It has been demonstrated that the transplantation of MSCs attenuates oxidative stress in in vitro models by upregulating the expression of the antioxidant enzymes SOD, CAT, GPx, and GSH [140]. In detail, MSCs efficiently manage oxidative stress-induced injury in vitro in neurons [141], renal cells [142], immune cells [143], islet cells [144], etc. Antioxidant effects have not only been reported in in vitro models; in MSC therapy, these effects have been reported through decreased oxidative stress markers and functional recovery in numerous disease models as follows: (1) Diabetic injury on the kidneys, retina, sensory neurons, brain, and bone formation; (2) chemotherapy- or radiation-induced injury on the lungs, gonads, aorta, and brain; (3) ischemic injury on the brain, heart, kidneys, and liver; (4) traumatic injury on the spine and testes, cognitive disorders, gastrointestinal inflammation, and septic injuries [145]. In particular, the transplantation of MSCs functions effectively in oxidative stress related to aging and female reproduction. Mouse BMSCs reduce lipid peroxidation and ameliorate oxidative stress in mitochondria in aged mice as a part of the antiaging mechanisms [146]. Mouse AMSCs decrease oxidative stress by reducing ROS levels and activating antioxidants, consequently inhibiting DNA damage in a premature aging mouse model [147]. In an aging-induced mouse model, mouse ASCs showed an antioxidative effect by regulating senescence-associated markers [148]. Mouse fetal liver-derived MSCs protected the POF mouse model from oxidative damage, contributing to the restoration of ovarian function and follicular development [149]. In the POF rat model, human umbilical cord-derived mesenchymal stem cells (UMSCs) restored ovarian function, connected with decreased autophagy-induced apoptosis and oxidative stress in theca-interstitial cells [150]. Not only in chemically-induced POF models but ovarian function was recovered by placenta-derived MSCs in an ovariectomized rat model due to the reduction of oxidative stress-induced apoptosis [151]. Most previous research has explored the effect of MSCs using direct transplantation, but the antioxidative effects of MSCs are also exerted in the paracrine method and, accordingly, the treatment of MSC-conditioned medium (CM) in recent years has resulted in the reduction of oxidative stress, suggesting the therapeutic effect of paracrine factors as an antioxidant defense mechanism [145].

## 5. Mesenchymal Stem Cell Conditioned Medium

A wide range of paracrine factors are secreted by MSCs, which are composed of an extracellular matrix, proteins involved in the adhesion process, enzymes and their activators and inhibitors, growth factors and binding proteins, cytokines, and chemokines [152]. These MSC-derived secreted factors are referred to as secretomes, microvesicles, or exosomes and are contained in the medium in which the cells are cultured, which is called the conditioned medium [153]. The presence of growth factors and other cytokines with regenerative effects in MSC-CM has been identified by protein detection analysis in various studies and are summarized by as follows: Vascular endothelial-derived growth factor, platelet-derived growth factor, epidermal growth factor, insulin like growth factor I and II, hepatocyte growth factor, fibroblast growth factor 2/basic fibroblast growth factor, keratinocyte growth factor/fibroblast growth factor 7, platelet-derived endothelial cell growth factor, heparin-binding epidermal growth factor, placenta growth factor, neural growth factor, and brain-derived neurotrophic factor; as inflammation-regulatory factors, TGF𝛽1, interleukin (IL)-10, IL-27, IL-17E, IL-13, IL-12p70, and IL-1 receptor antagonists, IL-8/CXCL-8, IL-9, and IL-1b; lastly, as other cytokines, leptin, angiogenin, granulocyte colony-stimulating factor (CSF), granulocyte macrophage CSF, macrophage CSF, fractalkine, monocyte chemotactic protein, serpin E-1, endostatin/collagen XVIII, UPA, thrombospondins 1 and 2, tissue inhibitor of metalloproteinase-1, IGF-binding protein stem cell-derived factor 1/CXCL-12, adrenomedullin, Dickkopf-1, and a few receptors [36].

Comparable therapeutic effects of its original cells on various diseases have been shown by MSC-CM, including myocardial infarction [154], stroke [155], spinal cord injury [156], brain injury [157], acute and chronic wounds [158], liver injury [159], kidney injury [160], periodontal injury [161,162], bone defects [163], musculoskeletal damage [164] skin disease [165] and male infertility [166]. Furthermore, clinical improvements from MSC-CM treatment have recently been reported in hair regeneration [167], inflammatory arthritis [168], and multiple sclerosis [169]. Above all therapeutic properties, accumulating studies have demonstrated the antioxidative effect of MSC-CM on in vitro and in vivo disease models [145]. Antioxidative ability has been displayed in MSC-CM from various types of MSCs, regardless of intra- or inter-specific treatment [145]. 

Especially in studies on reproduction, MSC-CM improves reproductive outcomes and exerts its antioxidative effects as follows: Human ASC-CM administered intravenously also improves mouse embryo development and implantation by increasing expression levels of antioxidant genes in reproductive organs against age-related infertility [170]. Not only can it be used as an in vivo treatment, but human ASC-CM can be employed as a supplement during in vitro culture, promoting mouse embryo developmental competence and reducing oxidative stress in developed embryos [171]. Along with ASC-CM, mouse preimplantation embryo development is enhanced, and intracellular oxidative stress is inhibited as the culture conditions improve after supplementation with human AMSC-CM as an antioxidant intervention [172]. The CM of human UCMSCs treated in in vitro maturation media increases the maturation and development of human oocytes, which shows promoted antioxidant gene expression as well [173]. Furthermore, intraperitoneal injection of human UCMSC-CM can alleviate follicle depletion against ovarian injury and upregulate gene expression, which suppresses oxidative stress-induced cell apoptosis in granulosa cells in mice [174]. Human UCMSC-CM injected peritoneally into a POF mouse model also protected injured ovaries and restored ovarian reserve by attenuating oxidative stress-induced apoptosis [174]. In addition to ovarian damage, mouse BMSC-CM supplemented during in vitro maturation (IVM) of oocytes from an endometriosis mouse model has been shown to enhance oocyte maturation and oxidative defense from nitric oxide, consequently improving fertilized embryo development [175]. The effect of MSC-CM is not restricted to humans and mice in that the treatment of equine amniotic fluid MSC-CM in porcine oocyte IVM media upregulates antioxidant enzymes in a culture environment and supports embryo development [176]. 

**Figure 2:**
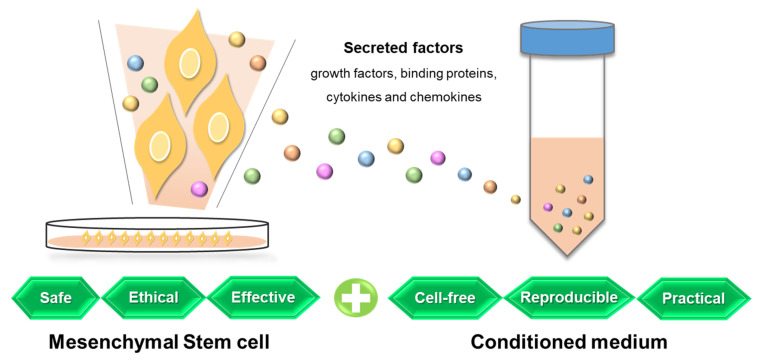
Representative advantages of mesenchymal stem cell conditioned medium, which contains secreted factors from source stem cells.

Promising potential has been shown by MSC-CM without the original cell from which it is derived, with reliability and reproducibility as a cell-free therapeutic, in that it can be easily manufactured, freeze-dried, packaged, and transported and does not need to match the donor and the recipient to avoid immune rejection [36] (Figure 2). In addition, compared to stem cells, MSC-CM possesses distinctive advantages with a lower production time and cost, a higher shelf life, controlled environmental conditions for application, and a sensitive storage method not being required [34]. Of note is that MSC-CM can be harvested from various types of cells and different culture conditions, which may regulate the level and function of secretory factors. Evaluation of MSC-CM from different sources has been conducted, demonstrating differences in the composition and subsequent effects [177,178,179,180,181,182,183]. For example, comparing CM from human ASCs and placental stem cells, the antiaging index improved more significantly after ASC-CM treatment [184]. In the field of assisted reproduction, human AMSC-CM is more effective than ASC-CM in supporting antioxidative conditions for mouse embryo development [172]. Interestingly, one study indicated that although human ASC-CM and BMSC-CM showed similar protective effects in rat hypoxia-induced injury, their action mechanisms were significantly different [185]. Even in the same type of cells, the level of growth factors can vary depending on culture conditions, number of cells, and concentration process used in the making of the MSC-CM [186,187,188]. Basal culture media could be one of the representative variables for the culture conditions of MSC-CM. It has been demonstrated that human ASC-CM based on Dulbecco′s modified Eagle′s medium is more antioxidative and antiapoptotic for mouse preimplantation embryo development than keratinocyte serum-free medium [171], which indicates that the basal medium used for MSC-CM collection can be a critical determinant of the capability of MSC-CM. As well as the production of MSC-CM, the treatment method of MSC-CM should be effectively considered to optimize its effectiveness. One study evaluated the efficacy of mouse ASC-CM in diabetic mice injected intravenously or intraperitoneally, which was more beneficial than systemic administration [189]. In addition, regarding the frequency of treatment, a higher frequency of human ASC-CM injections for aged female mice is more effective for embryo development and antioxidation [170]. Comprehensively, variables, including cell sources and protocols for production and application, need to be validated for the optimal effect of MSC-CM according to the purpose of its treatment. 

## 6. Conclusions

The decreased defense capacity of antioxidants, which occurs concurrently with aging, results in a loss of normal functions of the female reproductive system due to oxidative damage. Accordingly, assisted reproductive techniques need to be developed to solve infertility caused by reproductive aging and oxidative stress. The application of MSCs, including ASCs and AMSCs, in regenerative therapy, has been widely verified, with their strong antioxidant capacity. In addition to conventional cell therapy, the therapeutic and antioxidative efficacy of MSC-CM has been successfully reported to be as effective as the method of directly using the cells from which CM is derived, depending on the conditions of its production and application. In conclusion, further development of MSC-CM as a promising antioxidant is highly expected to improve assisted reproduction research, with its optimal treatment methods being confirmed.

## Figures and Tables

**Table 1:** Studies regarding the supplementation of antioxidant compounds to the embryo culture medium.

Antioxidants	Target	Effects of Treatment	Reference
α-tocopherol	Ovine embryos	↑ Cleavage rate ↑ Embryo developmental rate ↑ Blastocyst formation ↑ Total cell number of blastocyst	[104]
L-Carnitine	Mouse embryo	↑ Blastocyst development rate ↓ DNA damage	[105]
L-ergothioneine	Bovine embryos	↑ Blastocyst hatching rate ↑ ICM: total cells ratio ↓ Apoptotic cells	[106]
N-(2-mercaptopropionyl)-glycine	Porcine embryo	↑ Blastocyst formation ↓ Intracellular reactive oxygen ↓ Oxidative stress-related gene expression	[107]
Melatonin & Taurine	Buffalo embryo	↑ meiotic maturation rate ↑ Transferable embryo yield	[108]
SOD & Taurine	Feline embryo	↑ Oocyte maturation ↓ Oocyte degeneration ↑ Blastocyst number and quality	[109]
Vitamin C &E	Mouse embryo	↓ Embryo toxicity ↑ Blastocyst development rate	[110]

## Data Availability

Not applicable.
